# The malate synthase of *Paracoccidioides brasiliensis *is a linked surface protein that behaves as an anchorless adhesin

**DOI:** 10.1186/1471-2180-9-272

**Published:** 2009-12-24

**Authors:** Benedito Rodrigues da Silva Neto, Julhiany de Fátima da Silva, Maria José Soares Mendes-Giannini, Henrique Leonel Lenzi, Célia Maria de Almeida Soares, Maristela Pereira

**Affiliations:** 1 Laboratório de Biologia Molecular, Departamento de Bioquímica e Biologia Molecular, Instituto de Ciências Biológicas, Universidade Federal de Goiás, C.P. 131, 74001-970, Goiânia, GO, Brazil; 2 Laboratório de Micologia Clínica, Universidade Estadual Paulista, UNESP, Araraquara, SP, Brazil; 3 Laboratório de Patologia, Instituto Oswaldo Cruz-Fiocruz, Rio de Janeiro, RJ, Brazil

## Abstract

**Background:**

The pathogenic fungus *Paracoccidioides brasiliensis *is the agent of paracoccidioidomycosis (PCM). This is a pulmonary mycosis acquired by inhalation of fungal airborne propagules that can disseminate to several organs and tissues leading to a severe form of the disease. Adhesion and invasion to host cells are essential steps involved in the internalization and dissemination of pathogens. Inside the host, *P. brasiliensis *may use the glyoxylate cycle for intracellular survival.

**Results:**

Here, we provide evidence that the malate synthase of *P. brasiliensis *(*Pb*MLS) is located on the fungal cell surface, and is secreted. *Pb*MLS was overexpressed in *Escherichia coli*, and polyclonal antibody was obtained against this protein. By using Confocal Laser Scanning Microscopy, *Pb*MLS was detected in the cytoplasm and in the cell wall of the mother, but mainly of budding cells of the *P. brasiliensis *yeast phase. *Pb*MLSr and its respective polyclonal antibody produced against this protein inhibited the interaction of *P. brasiliensis *with *in vitro *cultured epithelial cells A549.

**Conclusion:**

These observations indicated that cell wall-associated *Pb*MLS could be mediating the binding of fungal cells to the host, thus contributing to the adhesion of fungus to host tissues and to the dissemination of infection, behaving as an anchorless adhesin.

## Background

Paracoccidioidomycosis (PCM), the most important systemic mycosis in Latin America, is a chronic granulomatous disease that affects about 10 million people.

*Paracoccidioides brasiliensis*, a thermally dimorphic fungus pathogen, is the pulmonary infective agent [1,2]. This initial interaction appears to govern the subsequent mechanisms of innate and acquire immunity, which result in localized infection or overt disease [3].

The mechanisms of adherence and invasion have been studied extensively in pathogenic bacteria [4], and in pathogenic fungi such as *Candida albicans *[5], *Histoplasma capsulatum *[6] and *Aspergillus fumigatus *[7], and *P. brasiliensis *[8-10]. Fungi are non-motile eukaryotes that depend on their adhesive properties for selective interaction with host cells [11]. Adherence molecules are fundamental in pathogen-host interaction; during this event, the fungal cell wall is in continual contact with the host and acts as a sieve and reservoir for molecules such as adhesins [12]. The ability of *P. brasiliensis *to adhere to and invade nonprofessional phagocytes or epithelial cells has been recognized in previous studies [13-15]. Some *P. brasiliensis *adhesins such as gp43 [10], glyceraldehyde-3-phosphate dehydrogenase (GAPDH) [16], a 30 kDa protein [9], and triosephosphate isomerase (TPI) [17] have been described. Evidence for extracellular localization of some glycolytic enzymes lacking secretion signals at cell-wall anchoring motifs has been reported for some pathogens [18,19]. In addition malate synthase (MLS) is also described as an adhesin on *Mycobacterium tuberculosis *[20].

The glyoxylate cycle and its key enzymes isocitrate lyase (ICL) and MLS play a crucial role in the pathogenicity and virulence of various fungi such as the human pathogens *A. fumigatus *[21], *Cryptococcus neoformans *[22] and *C. albicans *[23,24], the bacterium *M. tuberculosis *[25-27] as well as the phytopathogenic fungus *Magnaporthe grisea *[28] and the necrotropic wheat pathogen *Stagonospora nodorum *[29]. A relevant role for the glyoxylate cycle in the viability and growth of fungi inside macrophages and, consequently, in the development of a disseminated fungal infection has been postulated [21]. ICL and MLS have also been considered a therapeutic target for the development of novel antifungal compounds, since there are no human orthologues. In *P. brasiliensis*, the enzyme MLS (*Pb*MLS) participates in the glyoxylate pathway, which enables fungus to assimilate two-carbon compounds from the tricarboxylic acid cycle and in the allantoin degradation pathway of the purine metabolism, which allows the fungus to use nitrogen compounds [30].

Here it is demonstrated that *Pb*MLS is the first fungal MLS localized on the cell surface which interferes with the infection process.

## Results

### Expression, purification and production of polyclonal antibody to *Pb*MLSr

**Figure 1:**
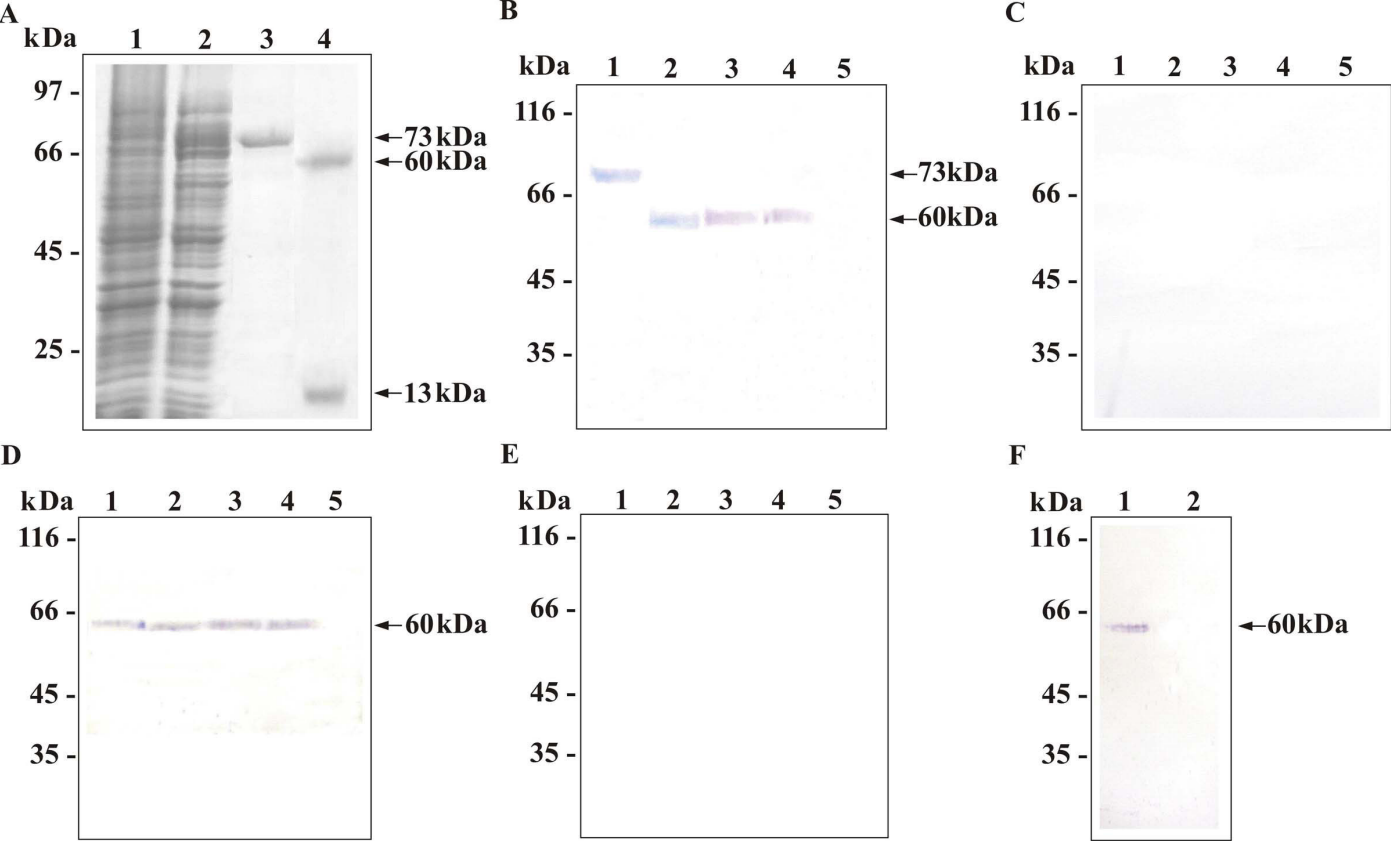
**Localization of *Pb *MLSr**. **(A) **SDS-PAGE analysis of *Pb*MLSr. *E. coli *BL21 C41 cells harboring the pET-32a-MLS plasmid were grown at 37°C to an OD_600 _of 0.6 and harvested before (lane 1) and after induction with 1 mM IPTG (lane 2). The cells were lysed by sonication, and the recombinant His-, Trx-, and S-Tagged *Pb*MLS were isolated by affinity chromatography (lane 3). Tags were removed by EKMax™ Enterokinase digestion (lane 4). **(B) **Western blots of fusion *Pb*MLSr (lane 1), cleaved *Pb*MLSr (lane 2), crude extract proteins from yeast cells (lane 3), SDS-extracted yeast cell wall proteins (lane 4), and yeast cell wall proteins (lane 5). Proteins were probed with anti-*Pb*MLSr antibody or with pre-immune rabbit **(C)**. **(D) **Western blots of proteins of culture filtrate of *P. brasiliensis *yeast cells harvested after 24 h (lane 1), 36 h (lane 2), 7 days (lane 3), and 14 days (lane 4) of culture, and culture filtrate without *P. brasiliensis *as negative control (lane 5). Proteins were probed with anti-*Pb*MLSr antibody or with pre-immune rabbit **(E)**. **(F) **Western blots of peroxisomal fraction (lane 1) and mitochondrial fraction (lane 2) proteins from *P. brasiliensis *yeast cells were probed with anti-*Pb*MLSr antibody. Molecular mass markers are indicated at the side.

The cDNA encoding *Pb*MLS was subcloned into the expression vector pET-32a to obtain recombinant fusion protein. The protein was not present in crude extracts of non-induced *E. coli *cells carrying the expression vector (Fig. 1A, lane 1). After induction with IPTG, a 73 kDa recombinant protein was detected in bacterial lysates (Fig. 1A, lane 2). The six-histidine residues fused to the N terminus of the recombinant protein were used to purify the protein from bacterial lysates by nickel-chelate affinity. The recombinant protein was eluted and analyzed by SDS-PAGE (Fig. 1A, lane 3) and His-, Trx-, and S-Tag were removed by cleavage with the enterokinase (Fig. 1A, lane 4). An aliquot of the purified recombinant protein was used to generate rabbit polyclonal anti-*Pb*MLSr antibody. Western blot confirmed the positive reaction of antibody with the fusion protein (Fig. 1B, lane 1) identifying a protein of 73 kDa. The cleaved recombinant protein was detected as a species of 60 kDa (Fig. 1B, lane 2).

### Detection of *Pb*MLS on cell wall extracts, culture filtrate, crude extract and peroxisomal fraction

To determine the subcellular distribution of *Pb*MLS, cell wall-enriched, secreted, and peroxisomal fractions purified from *P. brasiliensis *yeast cells were obtained. Crude extract proteins, SDS-extracted cell wall proteins, and extracted cell wall proteins from yeast cells were subjected to SDS-PAGE analysis, blotted onto nylon membrane and reacted to polyclonal anti-*Pb*MLSr antibody. *Pb*MLS was detected in crude extract (Fig. 1B, lane 3), and in SDS-extracted cell wall proteins (Fig. 1B, lane 4), but was not detected in extracted cell-wall proteins (Fig. 1B, lane 5). *Pb*MLS activity was evaluated in SDS-extracted cell wall and in crude extract, showing specific activity of 2131.2 U/mg and 2051.28 U/mg, respectively. No cross-reactivity to the pre-immune rabbit serum was evidenced with the samples (Fig. 1C).

To determined whether *Pb*MLS was secreted to the medium, proteins were extracted from culture filtrates harvested from *P. brasiliensis *which had been growing for 24 and 36 h (Fig. 1D, lanes 1 and 2, respectively), 7 days (Fig. 1D, lane 3), and 14 days (Fig. 1D, lane 4). The proteins were subjected to SDS-PAGE analysis, blotted onto nylon membrane and reacted to polyclonal anti-*Pb*MLSr antibody. *Pb*MLS was detected in all these preparations (Fig. 1D, lanes 1 to 4). No signal was detected in medium free of cells (Fig. 1D, lane 5). *Pb*MLS activity was evaluated in culture filtrate showing specific activity of 1305.3 U/mg. No cross-reactivity to the pre-immune rabbit serum was evidenced with the samples (Fig. 1E). Altogether, these results suggest that *Pb*MLS binds weakly to the cell wall and is actively secreted in *P. brasiliensis*.

Since *Pb*MLS has the AKL tripeptide, a peroxisomal/glyoxysomal signal which targets PTS1 [31], the presence of the protein was investigated in this cellular compartment. Peroxisomal and mitochondrial fractions purified of *P. brasiliensis *were obtained. The proteins were subjected to SDS-PAGE analysis, blotted onto nylon membrane and reacted to the polyclonal anti-*Pb*MLSr antibody. *Pb*MLS was detected in the peroxisomal fraction (Fig. 1F, lane 1) confirming the localization of *Pb*MLS in this organelle. Since *Pb*MLS has not been found in mitochondria, the mitochondrial fraction was used as the negative control (Fig. 1F, lane 2).

### Cellular localization of *Pb*MLS by confocal microscopy

**Figure 2:**
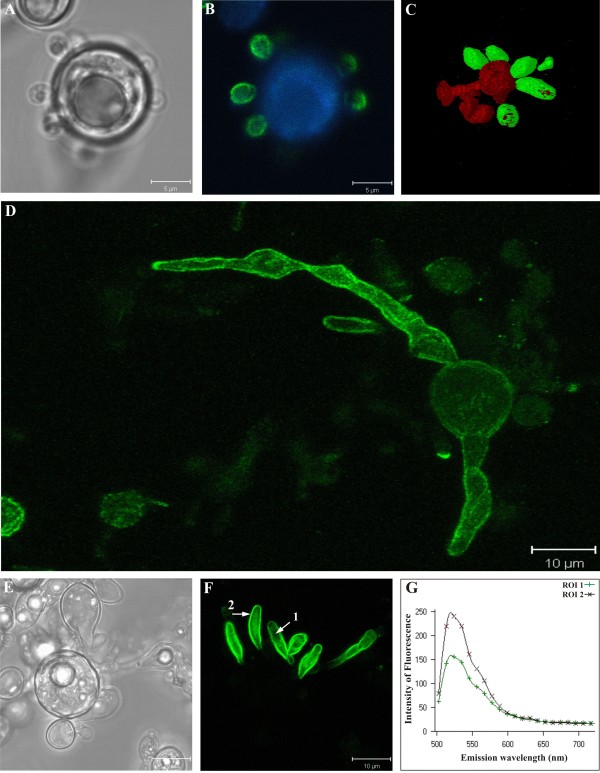
**Localization of *Pb *MLS by confocal laser scanning microscopy in *P. brasiliensis *yeast cells**. Differential accumulation of *Pb*MLS on the surface of budding cells is easily seen in **B, C **and **F**. Images **A **and **E **represent the differential interference contrast (DIC) of images **B **and **F**, respectively. Image **C **corresponds to a three-dimensional reconstruction of an immunofluorescent tomographic image showing the accumulation of *Pb*MLS only on the budding cells and not in the mother. This is also observed in images **B **and **F**. Image **G **displays the fluorescence pattern and intensity of two regions of interest (ROI) specified by arrows 1 and 2 in image **F**, indicating that the fluorescence is more intense on the cell surface (2) than in the cytoplasm of budding cells (1). Image **D **shows a mother cell positive to *Pb*MLS on the cellular surface and the formation, in culture, of budding cells also expressing *Pb*MLS.

To observe the cellular location of *Pb*MLS, *P. brasiliensis *yeast cells were grown in rich medium and visualized by laser confocal microscopy. The expression of *Pb*MLS was highly positive in the budding cells (Fig. 2 B, C and 2F) but was usually negative (Fig. 2 B and 2C) or weakly positive (Fig. 2 D) in the mother cells. Although reactivity was evident inside the cytoplasm of budding cells, it was much more intense on the cell surface (Fig. 2 F). The patterns and intensities of the fluorescence spectra of two regions of interest (ROI) are shown in Figure 2 G.

**Figure 3:**
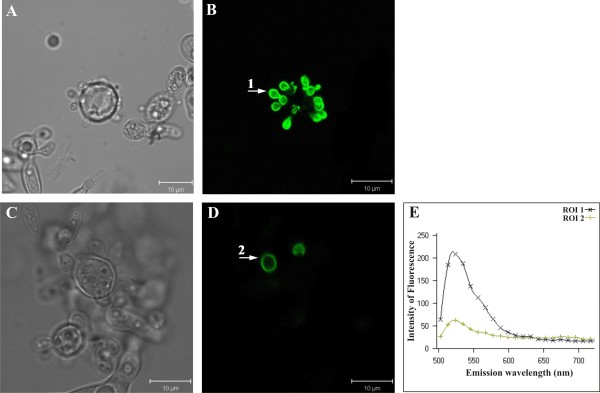
**Localization of *Pb *MLS by confocal laser scanning microscopy in *P. brasiliensis *yeast cells growing in different carbon sources**. The same groups of cells grown in the presence of potassium acetate (images **A **and **B**) or glucose (images **C **and **D**) as the sole carbon source are shown, side by side, using differential interference contrast microscopy (DIC) and confocal immunofluorescence. In both situations, the accumulation of *Pb*MLS was restricted to the budding cells. The graph in **E **displays, comparatively, the immunofluorescence patterns and intensities of two regions of interest (ROI 1 and 2), corresponding to arrows 1 and 2. The data indicate that, under the same labeling conditions, the budding cells cultivated on potassium acetate accumulate *Pb*MLS more intensely on the cell surface than those grown on glucose.

The localization of *Pb*MLS was also evaluated on *P. brasiliensis *yeast cells grown in medium containing acetate or glucose as the sole carbon source. Yeast cells accumulated *Pb*MLS in the presence of acetate (Fig. 3 B) or glucose (Fig. 3 D), but the quantity of *Pb*MLS was higher when the fungus was cultivated in the presence of acetate. This disparity was exemplified by the fluorescence spectra (Fig. 3 E), representative of two ROIs indicated by arrows 1 and 2 (Fig. 3 B and 3D). No cross reaction was observed with the pre-immune serum (data not shown).

### Binding of *Pb*MLSr to extracellular matrix proteins (ECM) and the reactivity to sera of PCM patients

**Figure 4:**
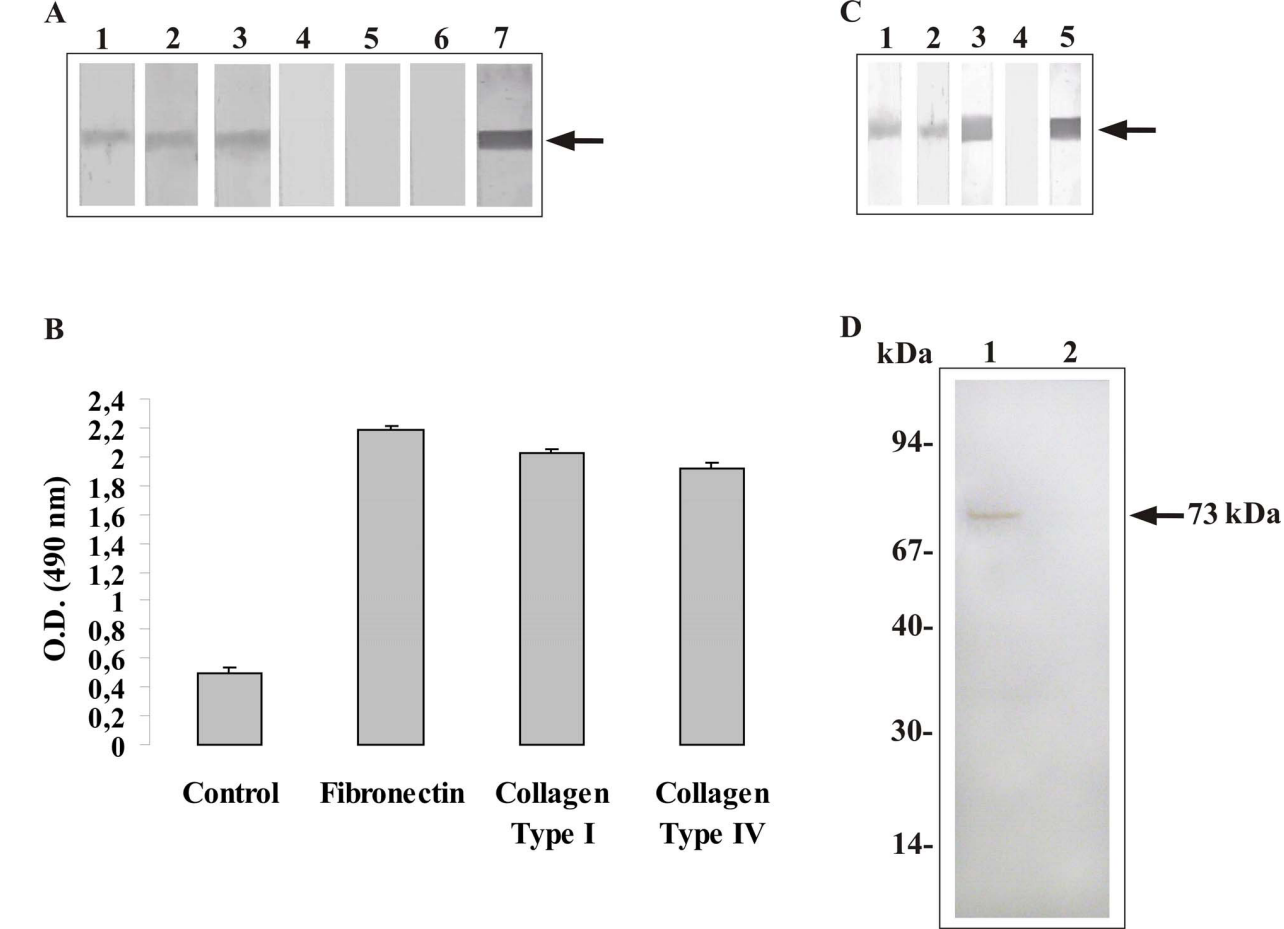
**(A) Binding of *Pb *MLSr to ECM by Far-Western blot**. *Pb*MLSr (0.5 μg) was subjected to SDS-PAGE and electroblotted. Membranes were reacted with fibronectin (lane 1), type I collagen (lane 2), type IV collagen (lane 3) and laminin (lane 4), and subsequently incubated with rabbit IgG anti-fibronectin, mouse anti-type I and anti-type IV collagen antibodies, and anti-laminin, respectively. Peroxidase-conjugated anti-rabbit and anti-mouse IgG revealed the reactions. Negative control was obtained by incubating *Pb*MLSr with peroxidase-conjugated anti-rabbit IgG (lane 5), and *Pb*MLSr with ECM (lane 6). Positive control was obtained by incubating *Pb*MLSr with polyclonal anti-*Pb*MLSr antibody (lane 7). **(B) **Binding of *Pb*MLSr to ECM fibronectin, types I and IV collagen (10 μg/mL). The interaction was revealed by ELISA with peroxidase-conjugated streptavidin. The results were expressed in absorbance units. The negative controls were performed using *Pb*MLSr only. **(C) **Reactivity of *Pb*MLSr to PCM patient sera. 1.0 μg of purified *Pb*MLSr was electrophoresed and reacted to the sera of patients with PCM, diluted 1:100 (lanes 1 to 3) and to control sera, diluted 1:100 (lane 4). The positive control was obtained by incubating *Pb*MLSr with its polyclonal antibody (lane 5). After reaction to the anti-human IgG alkaline phosphatase-coupled antibody (diluted 1:2000), the reaction was developed with BCIP-NBT. **(D) **Biotinylation assay by Western blot. Lysed A549 cells incubated with biotinylated *Pb*MLSr (lane 1); Lysed A549 cells (lane 2) as negative control.

The ability of the *Pb*MLSr to bind to ECM proteins was evaluated by Far-Western blot assays. *Pb*MLSr binds to fibronectin, type I and IV collagen, but not to laminin as shown in Fig. 4A, lanes 1, 2, 3 and 4, respectively). Negative controls were obtained incubating *Pb*MLSr with the secondary antibody in the absence of ECM or *Pb*MLSr with ECM only (Fig. 4A, lanes 5 and 6, respectively). The reaction between *Pb*MLSr and the antibody anti-*Pb*MLSr was used as a positive control (Fig. 4A, lane 7). The binding between *Pb*MLS and ECM compounds was also evaluated by ELISA assay. The results reinforced that *Pb*MLSr binds to fibronectin, type I and IV collagen (Fig. 4B). Negative controls were performed using *Pb*MLSr (Fig. 4B) or ECM only (data not shown). The positive control was performed using anti-*Pb*MLSr, anti-laminin, anti-fibronectin, anti-colagen I or anti-colagen IV antibody (data not shown).

*Pb*MLSr was reacted with three sera of patients with PCM and one serum from a healthy individual in immunoblot assays (Fig. 4C). Strong reactivity was observed with the PCM-patient sera (Fig. 4C, lanes 1 to 3). No cross-reactivity was observed with control serum (Figure 4C, lane 4). Reaction between *Pb*MLSr and anti-*Pb*MLSr was used as positive control (Fig. 4C, lane 5).

### Binding of *Pb*MLSr to pneumocytes

The ability of *Pb*MLSr to bind to A549 cells was evaluated. *Pb*MLSr was biotinylated and incubated with A549 cells. After lyses, proteins from A549 cells were electrophoresed by SDS-PAGE and blotted onto a membrane to perform Western blot with anti-*Pb*MLSr antibody. A positive signal was detected from lysed pulmonary A549 cells treated with biotinylated *Pb*MLSr (Fig. 4D, lane 1). The negative control was obtained using supernatant of A549 cells untreated with biotinylated protein (Fig. 4D, lane 2).

### The inhibitory effect of *Pb*MLSr and anti-*Pb*MLSr antibody on the interaction of *P. brasiliensis *cells with pneumocytes

**Figure 5:**
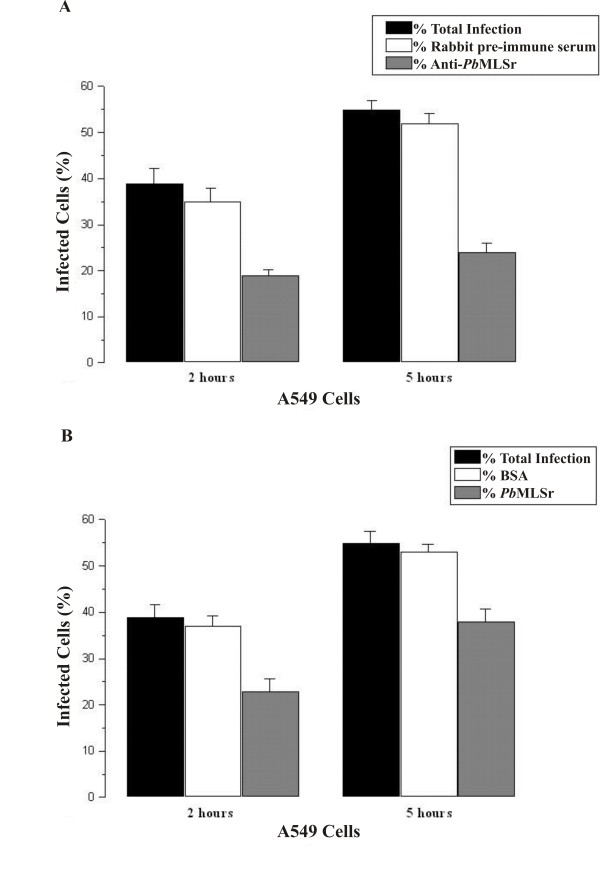
**Interaction of *P. brasiliensis *yeast forms with pneumocytes**. The interaction was assayed by indirect immunofluorescence and analyzed by flow cytometry. **(A) ***P. brasiliensis *yeast cells were pretreated for 1 h with anti-*Pb*MLSr polyclonal antibody (diluted 1:100), and control cells were pretreated with rabbit pre-immune serum. **(B) **A549 cells were pretreated for 1 h with 25 μg/mL of *Pb*MLSr, and control pneumocytes were pretreated for 1 h with 25 μg/mL of BSA. Adhesion of *P. brasiliensis *to pneumocytes was analyzed 2 h after the treatments. Infection (adhesion plus internalization) of *P. brasiliensis *to pneumocytes was analyzed 5 h after the treatments.

The infection index was determined by interactions between *P. brasiliensis *yeast cells and A549 pneumocytes, as shown in Figure 5. *P. brasiliensis *yeast cells were treated with the anti-*Pb*MLSr antibody before interaction with pneumocytes or pneumocytes were treated with *Pb*MLSr before interaction with *P. brasiliensis*. The controls (non-treated cells) were used to calculate the percentages of total infection. The interaction was analyzed by flow cytometry. Ten thousand events were collected to analysis as monoparametric histograms of log fluorescence and list mode data files. When *P. brasiliensis *yeast cells treated with anti-*Pb*MLSr antibody were incubated with A549 cells, a decrease in infection was observed after 2 h and 5 h of incubation (Fig. 5A). Similarly, after treatment of A549 cells with *Pb*MLSr, infection was reduced after 2 h and 5 h of incubation when compared to the values for non-treated cells (Fig. 5B). Controls were performed by incubating the pneumocytes with rabbit pre-immune serum or BSA before the addition of A549 cells or yeast cells (Fig. 5A and 5B, respectively).

## Discussion

Our studies showed that *Pb*MLS is a multifunctional protein; besides its enzymatic role as described by Zambuzzi-Carvalho [30], it could participate in the adherence process between the fungus and host cells through its ability to bind fibronectin, type I and type IV collagen. *Pb*MLS was detected in crude extract, cell wall and culture filtrate of *P. brasiliensis*, which is confirmed by activity assay. Taken together, our results suggest that *Pb*MLS is actively secreted by *P. brasiliensis*. In the same way, *M. tuberculosis *MLS has been consistently identified in the culture filtrates of mid-log phase *M. tuberculosis *cultures [32-34].

Adherence molecules are important in pathogen-host interactions. They operate as intercellular adhesion molecules (ICAM) or substrate adhesion molecules (SAM), contributing to cell-cell or cell-ECM adherences, respectively, and are usually exposed on the cellular surface. Successful host tissue colonization by fungus is a complex event, generally involving a ligand (adhesin) encoded by the pathogen and a cell or ECM receptor. The pathogen could interact with three types of host component: secreted cell products, host cell surface, or ECM proteins, such as types I and IV collagen, fibronectin, fibrinogen, and laminin [35]. The adhesin potential of *Pb*MLS was demonstrated through Far-Western blot, ELISA and binding assays. These showed that the recombinant protein recognized the ECM proteins, fibronectin and types I and IV collagen, as well as pulmonary epithelial cells. This event indicates that *Pb*MLS can play a role in the interaction of the fungus with host components. Studies have reported the capacity of *P. brasiliensis *for adhesion and invasion [9,15]. This is the first glyoxylate cycle enzyme identified on the fungal surface and released extracellularly which possesses the ability to bind to ECM proteins. The definition of *Pb*MLSr as a surface-exposed ECM-binding protein, with an unknown mechanism for secretion from the cell or sorting proteins to cellular membrane, suggests that *Pb*MLSr is compatible with anchorless adhesions [36,20]. In these types of adhesions, proteins are reassociated on the cellular surface after being secreted to execute their biological functions [36]. The presence of *Pb*MLS in the culture filtrate harvested after 24 and 36 h, and 7 and 14 days of growth confirmed that it is truly a secreted protein. The presence of *Pb*MLS in SDS-extracted cell-wall protein fraction indicates that *Pb*MLS is associated with the cell surface through weak interactions. Taken together these results provide evidence that *Pb*MLS may be transported out of the cell through the cell wall to be localized on the outer surface of the cell.

Reports have described the presence of some enzymes of the glycolytic pathway on the cell surface in *P. brasiliensis *as well as in other pathogens [16-19,37,38]. The presence of these housekeeping enzymes in unusual locations often correlates with their ability to perform alternative functions such as adherence/invasion of the host cells [38,18]. The ability of anti-adhesin antibodies to confer protection by blocking microbial attachment to host cells is being explored as a vaccination strategy in several microbial diseases [39-43]. The identification of the *Pb*MLS as a probable adhesin has several implications. Understanding the consequences of the binding of *Pb*MLS to host cells will lead to improved understanding of the initial events during infection. Further insights into the role of the *Pb*MLS in the host-pathogen interaction could contribute to the design of novel therapeutic strategies for PCM control.

Although PCM infection starts by inhalation of airborne propagules of the mycelia phase, as conidia, which reach the lungs and differentiates into the yeast phase [2], we performed experiments just with yeast cells since this is the phase found inside the host. Is important emphasize that *Pbmls *transcript is also present in the mycelium phase as described [44,45].

The results of confocal laser scanning microscopy demonstrated differences in the accumulation of *Pb*MLS among *P. brasiliensis *cells grown in different carbon sources. Accumulation of *Pb*MLS was also higher in *P. brasiliensis *yeast cells than in the mycelial phase (data not shown). These findings were reinforced by the results of Felipe *et al*. [44], which suggested that the glyoxylate cycle is up-regulated in yeast cells [46]. Yeast cells grown on potassium acetate accumulated more *Pb*MLS on the cell membrane than yeast cells grown on glucose. These results are in agreement with those obtained by Zambuzzi-Carvalho *et al*. [30] where the *Pbmls *transcript level was higher in yeasts cells grown in a two-carbon source than in cells grown on glucose only. The high intensity of ROI found in budding cells, mainly in the cellular membrane, suggests that the *Pb*MLS is metabolically relevant and mainly synthesized by young cells (budding cells). It is unknown whether *Pb*MLS plays any part in the differentiation and/or maturity processes of *P. brasiliensis *budding cells [45,47]. In fact, the glyoxylate pathway provides metabolic versatility for *Candida albicans *to utilize alternate substrata for development and differentiation and is involved in the formation of the filamentous state from the single cell state [23]. This process may help *Laccaria bicolor *grow toward the host with the aggressiveness required for mycorrhiza formation [48].

## Conclusion

The results showed the presence of *Pb*MLS in the culture filtrate of yeast cells (parasitic phase), its surface location in *P. brasiliensis *and its binding to ECM in Far-Western blot and ELISA assays and to A549 cells membranes. The reduction in the adherence of *P. brasiliensis *to A549 cells by anti-*Pb*MLSr suggests that *Pb*MLS could contribute to active fungal interaction and disease progression in humans through its ability to act as a probable adhesin. In addition, the absence of conventional secretion or cell wall anchoring motifs defines *Pb*MLS as a probable anchorless adhesin that could contribute to virulence by promoting *P. brasiliensis *infection and dissemination.

## Methods

### *P. brasiliensis *isolate and growth conditions

The *P. brasiliensis Pb*01 isolate (ATCC-MYA-826) was previously investigated in our laboratory and was cultivated in semisolid Fava Netto's medium (1.0% w/v peptone, 0.5% w/v yeast extract, 0.3% w/v proteose peptone, 0.5% w/v beef extract, 0.5% w/v NaCl, 4% w/v glucose and 1.4% w/v agar, pH 7.2) as yeast cells for 7 days at 36°C.

### Heterologous expression and purification of the *Pb*MLS recombinant (*Pb*MLSr)

The cDNA encoding to *Pb*MLS was obtained by Zambuzzi-Carvalho *et al*. [30] (GenBank accession number:AAQ75800). *Eco*RI and *Xho*I restriction sites were introduced in oligonucleotides to amplify a 1617 bp cDNA fragment of the *Pbmls*, which encodes a predicted protein of 539 amino acids. The PCR product was subcloned into the *Eco*RI/*Xho*I sites of the pET-32a(+) expression vector (Novagen, Inc., Madison, Wis.). The resulting plasmid was transferred to *Escherichia coli *BL21 C41 (DE3). Bacteria transformed with the pET-32a-MLS were grown in LB medium supplemented with ampicillim (100 μ/mL) at 37°C until reaching the optical density of 0.6 at 600 nm. Synthesis of the recombinant protein was then initiated by adding isopropyl-β-D-thiogalactopyranoside (IPTG) (Sigma-Aldrich, St. Louis, MO) to a final concentration of 1 mM to the growing culture and the bacterial extract was pelleted and resuspended in phosphate buffered saline (1 × PBS). After induction, the cells were incubated for 2 h at 37°C with shaking at 200 rpm. Cells were harvested by centrifugation at 10,000 × *g *for 5 min at 4°C. The supernatant was discarded and the cells were resuspended in 1 × PBS buffer. *E coli *cells were incubated for 60 min with lysozyme (100 μg/mL). After addition of 1% v/v Sarcosyl at 4°C, the cells were lysed by extensive sonication. The sample was centrifuged 8,000 × *g *for 15 min at 4°C and 2% v/v Triton was added to the supernatant containing the soluble protein fraction. His-tagged *Pb*MLSr was purified using the Ni-NTA Spin Kit (Qiagen Inc., Germantown, MD) and the tags were subsequently removed by the addition of EKMax™ Enterokinase (GIBCO™, Invitrogen, Carlsbad, CA).

### Antibody production

The purified *Pb*MLSr was used to produce anti-*Pb*MLSr polyclonal antibodies in New Zealand rabbits. The immunization protocol consisted of an initial injection of 300 μg of purified recombinant protein in complete Freund's adjuvant and two subsequent injections of the same amount of the antigen in incomplete Freund's adjuvant. Each immunization was followed by an interval of 14 days. After the fourth immunization, the serum containing the anti-*Pb*MLSr polyclonal antibody was collected and stored at -20°C.

### Western blotting analysis

SDS-PAGE was performed in 12% polyacrylamide gels according to Laemmli [49]. The proteins were electrophoresed and stained with Coomassie brilliant blue or transferred to a nylon membrane and checked with Ponceau S to determine equal loading. *Pb*MLS, as well as *Pb*MLSr, were detected with the polyclonal antibody raised against the recombinant protein (diluted 1: 4000). After reaction with alkaline phosphatase anti-mouse immunoglobulin G (IgG) or alkaline phosphatase anti-human IgG, the reaction was developed with 5-bromo-4-chloro-3-indolylphosphate-nitroblue tetrazolium (BCIP-NBT).

### Cell wall protein extractions

Yeast cells were frozen in liquid nitrogen and disrupted using a mortar and pestle. The procedure was carried out until complete cell rupture, verified by microscopic analysis, and by the failure of cells to grow on Fava Netto's medium. Ground material was lyophilized and resuspended in 25 μL Tris buffer (50 mM Tris-HCl, pH 7.8) for each milligram of dry weight, as previously described [50]. The supernatant was separated from the cell wall fraction by centrifugation at 10,000 × *g *for 10 min at 4°C. The crude extract was kept and a new protein extraction was performed with the Tris buffer as described above. The cell wall was extensively washed in solutions with decreasing concentrations of 1 M NaCl to remove any extracellular or cytosolic protein contaminants that could adhere to the walls through electrostatic forces. Isolated cell walls were treated with SDS-extraction buffer (50 mM Tris-HCl, pH 7.8, 2% w/v SDS, 100 mM Na-EDTA, and 40 mM *β*-mercaptoethanol) to extract cell surface-associated proteins, i.e. proteins loosely associated with the cell surface through non-covalent interactions or disulfide bridges (SDS-SW). The proteins from the cell wall and from crude extract were quantified according to Bradford [51].

### Preparation of culture filtrate proteins

The culture filtrate were processed as described previously [52], with modifications. Briefly, after 24 and 36 h, and 7 and 14 days of growth at 37°C with gentle agitation, the culture supernatant were removed from the cells by filtration and the culture filtrate was dialyzed and dried by lyophilization. The protein content of the concentrated culture filtrate was quantified according to Bradford [51].

### Preparation of Peroxisomal Fraction

The Peroxisome Isolation Kit (Sigma-Aldrich) was used in the preparation of crude peroxisomal fraction from cell cultures *P. brasiliensis Pb*01 (~2 × 10^8 ^cells) by differential centrifugation followed by density gradient centrifugation. Briefly, spheroplasts were obtained at 30°C by lysing the cell wall in 400 U of lyticase (Sigma) for 24 h. Spheroplast membranes were disrupted using a grinder and pestle. After centrifugation for 10 min, the crude peroxisomal fraction was obtained. The organelles were isolated by density gradient centrifugation to separate the enriched peroxisomes fraction from the purified mitochondrial fraction using the Peroxisome Isolation Kit.

The presence of peroxisomes was determined by measuring the activity of the peroxisomal enzyme marker catalase (Catalase Assay Kit) (Sigma-Aldrich). Separation of peroxisomes from mitochondria was determined by measuring the activity of the mitochondrial enzyme marker, cytochrome c oxidase (Cytochrome c Oxidase Assay Kit) (Sigma-Aldrich). In addition, peroxisomal membrane proteins were detected and their degree of enrichment in the purified fraction was determined by immunoblot using anti-*Pb*MLSr.

### Affinity ligand assays

Far-Western blot assays were carried out as previously described [53]. *Pb*MLSr underwent SDS-PAGE and was blotted onto nylon membrane. Blotted protein was assayed for laminin, fibronectin, type I and type IV collagen, or for PCM patients' sera as follows. After being blocked for 4 h with 1.5% (w/v) BSA in 10 mM PBS-milk and then washed three times (for 10 min each time) in 10 mM PBS-T, the membranes were incubated with laminin (20 μg/mL), fibronectin (20 μg/mL), or type I and IVcollagen (30 μg/mL), diluted in PBS-T with 2% BSA for 90 min, and then washed three times (for 10 min each time) in PBS-T. The membranes were incubated for 18 h with rabbit anti-laminin, anti-fibronectin, anti-type I collagen or anti-type IV collagen antibodies in PBS-T with 2% BSA (diluted 1:100). The blots were washed with PBS-T and incubated with peroxidase-labeled goat anti-rabbit immunoglobulin (diluted 1:1000). The blots were washed with PBS-T and the reactive signals were developed with hydrogen peroxide and diaminobenzidine (Sigma-Aldrich) as the chromogenic reagent. The positive control was obtained by incubating the *Pb*MLSr with the polyclonal anti-*Pb*MLSr antibody (diluted 1:500), and the reaction was developed as described above.

### ELISA analysis

ELISA was carried out as previously described by Mendes-Giannini *et al*. [8] with modifications. Briefly, Polypropylene 96-well microtiter ELISA plates were sensitized with extracellular matrix (ECM) proteins (10 μg/mL), overnight at 4°C. After blocking with 2% w/v BSA, 10% v/v SFB and 1% w/v milk, the incubation was followed with *Pb*MLSr (5 μg/mL) for 2 h at 37°C in triplicate wells. The reaction was developed using buffer citrate pH 4.9 conjugated with o-phenylenediamine as chromogenic substrate. Negative controls were performed using *Pb*MLSr or ECM only. Positive controls were performed using anti-*Pb*MLSr, anti-laminin, anti-fibronectin, anti-colagen I or anti-colagen IV antibody. The absorbance was measured at 490 nm and the results were analyzed by using Software Microcal ™Origin ™ software version 5.0 Copyright^© ^[54].

### Inhibition assay of *P. brasiliensis *interaction with epithelial cells using *Pb*MLSr and anti-*Pb*MLSr antibody

A549 pneumocytes were incubated for 1 h at 37°C with *Pb*MLSr (50 μg/mL), diluted in 10 mM PBS. After this incubation period, the cells were washed three times in PBS and 10^6 ^yeast forms of *P. brasiliensis *were added. Incubation was performed for 2 and 5 h at 37°C to allow invasion and internalization, respectively, as described previously [9,15,13]. Four control experiments were performed using A549 cells not preincubated with *Pb*MLSr; *P. brasiliensis *yeast cells not preincubated with the anti-*Pb*MLSr antibody; pneumocytes preincubated with BSA (25 μg/mL) and *P. brasiliensis *yeast cells preincubated with rabbit pre-immune serum. The percentage of infected cells was obtained by flow cytometry (BD FACSCanto) (BD Biosciences, Hialeah, FL). An adhesion index was created by multiplying the mean number of attached yeast cells per pneumocyte by the percentage of infected cells. The infection index (adherence plus internalization) was determined by the number of total fungi interacting with the epithelial cells 5 h after addition of the yeast cells, as previously described [15,13]. The mean and S.D. of at least three independent experiments were determined. Statistical analysis was calculated by using ANOVA (*F *test followed by Duncan test). *P *values of 0.05 or less were considered statistically significant.

### Biotinylation of protein

*Pb*MLSr was biotinylated with the ECL protein biotinylation kit (GE Healthcare, Amersham Biosciences) as recommended by the manufacturer. Monolayers of A549 cells were incubated with the biotinylated proteins at 37°C overnight and washed with PBS to remove unbound protein. Next, double-distilled water was added and the cells were incubated for 4 h at 25°C to obtain total lysis. The lysates were centrifuged at 1,400 × *g *for 5 min, and the supernatant underwent electrophoresis by SDS-PAGE. Proteins in the gel were blotted onto a nylon membrane; membrane strips were incubated with blocking buffer for 4 h at 25°C. Incubation for 1 h with streptavidin-HRP followed. A control containing *Pb*MLSr was revealed with the Catalyzed Signal Amplification (CSA) System kit (DAKO). The negative control was developed with the supernatant of A549 cells after lyses (without incubation with the biotinylated protein).

### Confocal analysis

The cellular localization of the *Pb*MLS was performed as described by Batista *et al*. [55] and Lenzi *et al*. [56] for confocal laser scanning microscopy (CLSM). Briefly, the cells growing in different sources of carbon were fixed in 4% paraformaldehyde for 1 h, washed and centrifuged. After permeabilization with Triton X-100, the cells were washed in PBS and incubated in blocking solution (2.5% BSA, 1% skim milk, 8% fetal calf serum) for 20 min (Fernandes da Silva, 1988). The diluted (1:100) primary antibody anti-*Pb*MLSr was added overnight at 4°C. After washing three times with PBS, the cells were incubated with secondary antibody (Alexa Fluor 488 anti-rabbit Molecular Probes 1:700) for 1 hour. Before mounting in 90% glycerol in PBS, adjusted to pH 8.5, containing antifading agent (p-phenylenediamine 1 g/L) (Sigma-Aldrich), the cells were stained with Evans blue (1/10000 in 0.01 M PBS). The specimens were analyzed by laser confocal microscopy (LSM 510-META, Zeiss).

### Flow cytometry assay analysis

All flow cytometry analyses were performed on a BD FACSCanto (BD Biosciences) using an air-cooled argon-ion laser tuned to 488 nm and 115 mW. The flow rate was kept at approximately 10,000 events (cells), and green fluorescence was amplified logarithmically. Ten thousand events were collected as monoparametric histograms of log fluorescence, as well as list mode data files. The data were analyzed by FACSDiva Software (BD Biosciences) and Origin Software [54].

### Enzymatic activity

MLS activity was determined as described by Zambuzzi-Carvalho (2009) [30]. Briefly, the enzymatic assay was carried out at room temperature. 25 mg samples were added to 500 ml assay buffer containing 5 mM acetyl-CoA (20 ml) and water to a volume of 980 ml. The reaction had the optical densities read at 232 nm until stabilization. The enzymatic reaction was started by the addition of 100 mM glyoxylate (20 ml). The method is based on the consumption of acetyl-CoA at 232 nm. The activity was calculated considering that one unit at 232 nm is defined as 222 nmols/mg of acetyl-CoA. The specific activities were given in U/mg protein, with U being equal at nmol/min.

### Statistical analysis

Results are expressed as the mean ± SD of the mean of three independent experiments. Statistical analysis was performed using ANOVA (F-test followed by Duncan test). P-values of 0.05 or less were considered statistically significant.

## Authors' contributions

BRSN carried out all assays. JFS and MJSMG participated in the adhesion and infection assays. HLL participated in confocal assays. BRSN, MJSMG, HLL, CMAS and MP contributed to the preparation of the manuscript. MP conceived, designed and coordinated the study. All authors contributed to the discussion of results. All the authors have read and approved the final manuscript.
